# Inhibition of NF-κB in Tumor Cells Exacerbates Immune Cell Activation Following Photodynamic Therapy

**DOI:** 10.3390/ijms160819960

**Published:** 2015-08-21

**Authors:** Mans Broekgaarden, Milan Kos, Freek A. Jurg, Adriaan A. van Beek, Thomas M. van Gulik, Michal Heger

**Affiliations:** 1 Department of Experimental Surgery, Academic Medical Center, University of Amsterdam, 1105 AZ Amsterdam, The Netherlands; E-Mails: mbroekgaarden@mgh.harvard.edu (M.B.); milan.kos@amc.uva.nl (M.K.); freek@moonshots.nl (F.A.J.); t.m.vangulik@amc.uva.nl (T.M.G.); 2 Department of Cell Biology and Immunology, Wageningen University, 6709 PG Wageningen, The Netherlands; E-Mail: adriaan.vanbeek@wur.nl

**Keywords:** phototherapy, inflammation, anti-tumor immunity, siRNA, interleukin-6, tumor necrosis factor alpha, monocyte chemotactic protein 1 interleukin-10, interleukin-12p70, interferon gamma

## Abstract

Although photodynamic therapy (PDT) yields very good outcomes in numerous types of superficial solid cancers, some tumors respond suboptimally to PDT. Novel treatment strategies are therefore needed to enhance the efficacy in these therapy-resistant tumors. One of these strategies is to combine PDT with inhibitors of PDT-induced survival pathways. In this respect, the transcription factor nuclear factor κB (NF-κB) has been identified as a potential pharmacological target, albeit inhibition of NF-κB may concurrently dampen the subsequent anti-tumor immune response required for complete tumor eradication and abscopal effects. In contrast to these postulations, this study demonstrated that siRNA knockdown of NF-κB in murine breast carcinoma (EMT-6) cells increased survival signaling in these cells and exacerbated the inflammatory response in murine RAW 264.7 macrophages. These results suggest a pro-death and immunosuppressive role of NF-κB in PDT-treated cells that concurs with a hyperstimulated immune response in innate immune cells.

## 1. Introduction

Photodynamic therapy (PDT) is a treatment modality for solid tumors in which the tumor is photosensitized following administration of a photosensitizer and subsequently irradiated with (laser) light at resonant frequencies (typically in the red wavelength range). Excitation of the photosensitizer results in the photochemical production of reactive molecular species that oxidize biomolecules, leading to the demise of tumor- and tumor-associated cells and ultimately removal of the tumor [1]. Although PDT is associated with good clinical outcomes in superficial cancers [2], there is a strong medical need to improve therapeutic efficacy in cancer patients where PDT is employed as a last-line treatment, including non-resectable hilar cholangiocarcinoma [2,3]. Current approaches to improve PDT are focused on new photosensitizing agents [4], the development of targeted photosensitizer delivery systems [5,6], as well as identification of adjuvant chemotherapeutics [7,8] and inhibitors of tumor survival pathways [2].

PDT-related survival pathways include the nuclear factor E2-related factor 2 (NRF2), nuclear factor κB (NF-κB), hypoxia inducible factor 1 (HIF-1), heat shock factor 1 (HSF-1), and apoptosis signal regulating kinase 1 (ASK-1) signaling pathways, as well as the proteotoxic stress response (reviewed in [2]). However, the role of NF-κB in post-PDT survival is equivocal. Its inhibition in relation to PDT efficacy has been sporadically investigated. Although there are only few investigations towards inhibition of NF-κB in relation to PDT efficacy, NF-κB is generally considered to mediate pro-survival and anti-apoptosis signaling following PDT [9,10], albeit some studies have observed pro-apoptotic effects following NF-κB activation [11]. Moreover, the genes regulated by the κB promoter element include cytokines, growth factors, and angiogenic proteins [12,13,14]. PDT-induced alterations in NF-κB signaling, which is partly under redox control [15], may therefore translate to perturbations in immune cell activation and an anti-tumor immune response [2,16].

The anti-tumor immune response is a critical element in tumor eradication. The direct cytotoxic effects of PDT-induced hyperoxidative stress are therapeutically inadequate, as treated tumors tend to recur in the absence of an intact immune system [17,18] and abscopal clearance of non-afflicted tumor cells is required for a cancer-free state [19,20]. The interconnectedness between post-PDT biochemical signaling cascades and corollary biological responses is further convoluted by the “unconventional” and complex cellular landscape following PDT [21].

Although the pharmacological inhibition of NF-κB signaling may increase PDT efficacy [22], the effects on the therapeutically important anti-tumor immune response [23] remain elusive. This study therefore investigated the role of NF-κB in the survival of EMT-6 murine mammary carcinoma cells after PDT with newly developed liposomes containing the second-generation photosensitizer zinc phthalocyanine and the effect of PDT-treated tumor cells on innate immune cell (RAW 264.7 macrophage) activation and inflammatory signaling.

## 2. Results

### 2.1. Photodynamic Therapy (PDT)-Killed EMT-6 Cells Are Immunogenic

PDT was performed using the photosensitizer zinc phthalocyanine (ZnPC) encapsulated in PEGylated cationic liposomes intended to target tumor endothelium and tumor cells *in vivo* (referred to as endothelium-targeted liposomes or “ETLs”) [6,8]. ZnPC (logP of ~8) was encapsulated into liposomes to render the photosensitizer compatible with blood and to enable selective targeting to pharmacologically important locations in the tumor. These liposomes are termed ZnPC-encapsulating endothelium-targeted liposomes or “ZnPC-ETLs” [24].In other studies we demonstrated that ZnPC-ETLs were stable over a period of seven days [25] and produced reactive oxygen species upon irradiation that oxidized the redox probe 2′,7′-dichlorodihydrofluorescein (DCFH_2_) [26] and proteins [24]. ZnPC in ETLs was more effective in oxidizing substrates than the ZnPC in neutral liposomes [24]. Moreover, the target cells took up these liposomes *in vitro*, and induced lethal cytotoxicity upon PDT at low-nM IC_50_ values [24]. The ZnPC-ETLs were therefore investigated in this study in the context of survival- and immune signaling, as these are important determinants of PDT outcome [2,5,23].

**Figure 1:**
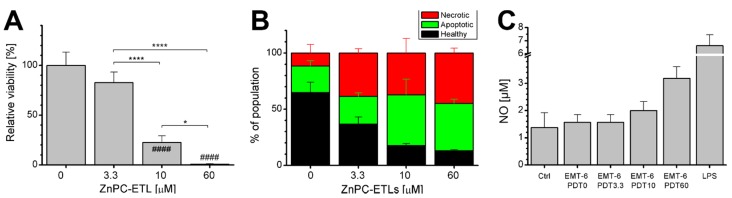

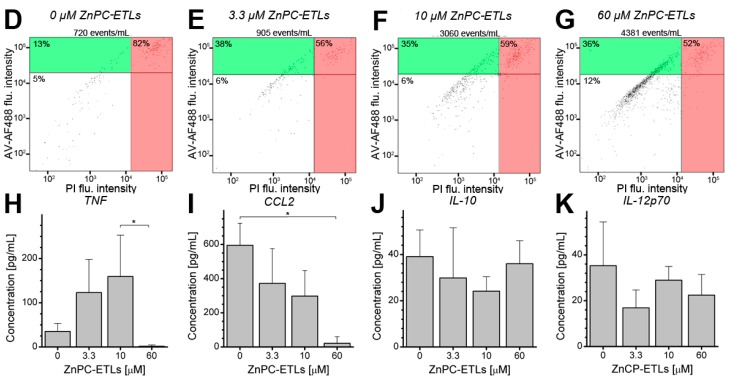
(**A**) Viability of EMT-6 cells as determined 24 h after photodynamic therapy (PDT) with zinc phthalocyanine-encapsulating endothelium-targeted liposomes (ZnPC-ETLs) at the indicated concentrations. Cells were placed in hypoxic conditions immediately following PDT (mean ± SD, *N* = 6). Data were analyzed using a one-way ANOVA and Sidak’s *post-hoc* test. Significant differences *versus* the corresponding control group are indicated with a pound sign, whereas are indicated with asterisks. The number of signs indicates the level of significance: * *p* < 0.05, **** *p* < 0.001 for intergroup differences, #### *p* < 0.001 *versus* control; (**B**) Flow cytometry-based characterization of the mode of EMT-6 cell death 24 h after PDT and subsequent incubation under hypoxic conditions (mean ± SD, *N* = 3). Staining was performed with Alexa Fluor 488-conjugated annexin V (apoptosis) and propidium iodide (necrosis); (**C**) The medium from EMT-6 cells was harvested 24 h after PDT and subsequent hypoxic incubation, and added to cultured RAW 264.7 macrophages. After 24 h of stimulation, macrophage activation was assessed by measuring nitric oxide (NO) production, which is a measure of macrophage activation. Cells were stimulated with 1 μg/mL lipopolysaccharide (LPS) for 24 h as positive control. The number behind “PDT” refers to the final lipid concentration of ZnPC-ETLs; (**D**–**G**) The medium from PDT-treated EMT-6 cells was analyzed for healthy, apoptotic (green), and necrotic (red) cells by flow cytometry; (**H**–**K**) Cytokine release by PDT-treated EMT-6 cells was assayed 24 h after PDT and subsequent hypoxic incubation (mean ± SD, *N* = 3). The “0 μM ZnPC-ETLs” group was not irradiated and served as negative control. Data were analyzed using a Kruskal–Wallis test and Dunn’s *post-hoc* test for multiple comparisons. The ZnPC-ETL concentrations (*x*-axes) refer to final lipid concentration. The ZnPC:lipid molar ratio was 0.003 [6]. * *p* < 0.05.

First, the PDT efficacy of ZnPC-ETLs was determined in EMT-6 cells at 24 h post-PDT and subsequent hypoxic incubation, showing a significant photosensitizer dose-dependent decrease in viability (Figure 1A). Flow cytometric analysis performed 24 h post-PDT revealed that tumor cell death occurred predominantly via apoptosis and potentially secondary necrosis (Figure 1B). The addition of PDT-treated EMT-6 cell supernatant to subconfluent RAW 264.7 macrophages caused an increase in nitric oxide (NO) production (Figure 1C), which is a hallmark of macrophage activation [27]. The supernatant of PDT-subjected EMT-6 cells contained viable, apoptotic, and necrotic cells. The quantity of the supernatant-suspended cells increased in a ZnPC concentration-dependent manner (Figure 1D–G), which was inversely proportional to post-PDT cell viability (Figure 1A). Moreover, the supernatant of EMT-6 cells subjected to PDT at the highest ZnPC dose induced the most extensive macrophage activation (Figure 1C), demonstrating that PDT triggers innate immune cell activation in proportion to the degree of tumor cell injury.

### 2.2. PDT-Killed EMT-6 Supernatant Contains Tumor Necrosis Factor α (TNF-α) and Chemokine C–C Motif Ligand 2 (CCL2)

The supernatant of PDT-treated EMT-6 cells contained high levels of tumor necrosis factor α (TNF-α) and chemokine C–C motif ligand 2 (CCL2, also referred to as monocyte chemotactic protein or MCP-1), and low levels of interleukin (IL) 10 and IL-12p70 (Figure 1H–K). The irradiated EMT-6 cells appear to have actively released TNF-α and CCL2 following PDT, as opposed to passive efflux from dead cells, given that the cytokines were significantly lower in the supernatant of EMT-6 cells that had been subjected to PDT at the highest photosensitizer concentration (Figure 1H,I), which contained a minimum of viable cells (Figure 1A,B). It is unlikely that PDT caused oxidative destruction of the cytokines given the inversely proportional relationship between TNF-α release and phototoxicity (Figure 1H *versus* A) in the 0–10 μM concentration range. The TNF-α-versus-viability trend suggests that this cytokine may be strongly upregulated as part of post-PDT survival signaling [2]. The release of CCL2 was proportional to the damage profile (Figure 1I), whereas the release of IL-10 and IL-12p70 was unaffected by PDT (Figure 1J,K). The expression of IL-6 and interferon γ (IFN-γ) did not exceed the limit of detection (not shown). The supernatant that contained the lowest amount of cytokines (the 60-μM ZnPC-ETL group) was the most immunogenic (Figure 1C), suggesting that other factors in the supernatant caused immunogenicity. Given the amount of cell debris in this medium (non-gated region in the flow cytograms, not shown), the extensive immune cell activation was most likely caused by non-assayed constituents such as damage-associated molecular patterns (DAMPs) [5,28,29].

### 2.3. Inhibition of Nuclear Factor κB (NF-κB) Reduces Cell Death

**Figure 2:**
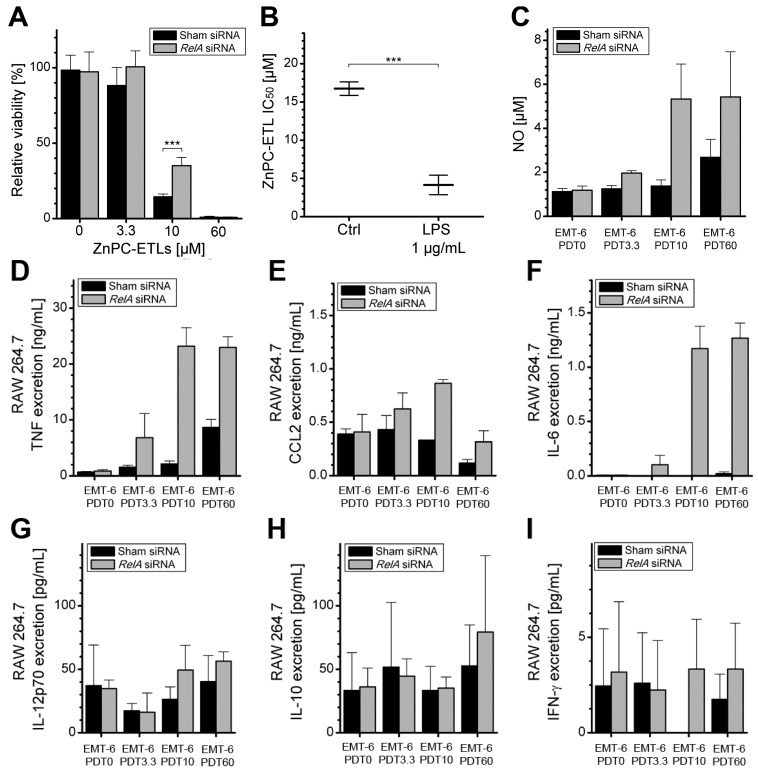
(**A**) EMT-6 cells were treated with either sham siRNA (black bars) or *RelA* siRNA (grey bars) and subsequently subjected to PDT with increasing concentrations of ZnPC-ETLs (indicated by the number after “PDT”). Cell viability was assessed 24 h after PDT and subsequent hypoxic incubation (mean ± SD, *N* = 6, student’s *t*-test). *** *p* < 0.005; (**B**) ZnPC-ETL IC_50_ values as determined on EMT-6 cells that were preconditioned with LPS *versus* non-preconditioned cells. *** *p* < 0.005; (**C**) NO production by RAW 264.7 macrophages 24 h after stimulation with supernatant from PDT-treated EMT-6 or EMT-6-*RelA*^kd^ cells (mean ± SD, *N* = 3); (**D**–**I**) After 24 h of incubation with supernatant from PDT-treated EMT-6 or EMT-6-*RelA*^kd^ cells, the medium from the RAW 264.7 cells was assayed for cytokine levels (means ± SD, *N* = 3). The ZnPC-ETL concentrations (*x*-axes) refer to final lipid concentration (indicated by the number after “PDT”). The ZnPC:lipid molar ratio was 0.003 [6].

Given that NF-κB mediates cell survival [9,10] as well as transcriptional upregulation and synthesis of the assayed cytokines [2,13], it was hypothesized that inhibition of NF-κB in EMT-6 cells would improve PDT efficacy and reduce the pro-inflammatory signaling by PDT-afflicted tumor cells. Accordingly, the mRNA that encodes RelA (reticuloendotheliosis A, nuclear factor NF-κB p65 subunit) was knocked down with siRNA (designated as “EMT-6-*RelA*^kd^”), thereby abrogating the activity of an essential subunit of the NF-κB transcription factors [30]. Control cells were transfected with sham siRNA. The EMT-6-*RelA*^kd^ cells were subsequently subjected to the same PDT protocol as used in the previous assays (Figure 1). EMT-6-*RelA*^kd^ cells were more resistant to PDT-induced cell death (Figure 2A), indicating that NF-κB plays a pivotal role in the promotion of EMT-6 tumor cell death following PDT. As NF-κB can be activated with lipopolysaccharide (LPS) [31,32], the results were corroborated in positive control experiments. Tumor cells pretreated with LPS were significantly more susceptible to PDT compared to non-pretreated cells (Figure 2B).

### 2.4. Increased Immunogenicity of PDT-Killed EMT-6-RelA^kd^ Cells

In addition to reduced extents of EMT-6 cell death caused by *RelA* knockdown, the supernatant obtained from the EMT-6-*RelA*^kd^ cells was more immunogenic compared to that of wild type cells, as evidenced by greater extent of NO production by RAW 264.7 cells (Figure 2C *versus* Figure 1C). A substantial increase in TNF-α levels and a moderate increase in CCL2 levels were detected following incubation of RAW 264.7 cells with the supernatant derived from PDT-treated EMT-6-*RelA*^kd^ cells (Figure 2D,E). Moreover, the knockdown of RelA considerably potentiated IL-6 release from the stimulated macrophages (Figure 2F), which was absent in macrophages primed with the supernatant of irradiated sham-transfected EMT-6 cells. Although low levels of IL-12p70, IL-10, and IFN-γ were detected in the medium of stimulated RAW 264.7 macrophages (Figure 2G–I), there were no notable intergroup differences.

## 3. Discussion

Several key findings were made in this study, namely (1) PDT-treated tumor cells release pro-inflammatory cytokines (TNF-α and CCL2) up to a certain phototoxic damage threshold; (2) the supernatant of PDT-treated tumor cells activates macrophages in proportion to the extent of tumor cell death; (3) this macrophage activation occurs via the tumor cell-derived cytokines as well as other immunogenic constituents in the supernatant of PDT-treated tumor cells; (4) activated macrophages exacerbate pro-inflammatory signaling by secreting TNF-α, CCL2, and IL-6; (5) PDT-induced tumor cell death is promoted by NF-κB; and (6) inhibition of NF-κB in tumor cells confers an intensified pro-inflammatory response in macrophages primed with tumor cell-derived supernatant, with substantial increases in IL-6 secretion.

The results in Figure 1 demonstrate that the immunogenicity of dying tumor cells is most likely caused by DAMP release from the apoptotic and necrotic tumor cells, given the absence of prevalent inflammatory cytokines in the supernatant of cells treated with the highest photosensitizer dose. DAMPs that are commonly released after PDT are heat shock proteins and high mobility group protein 1 [5,28]. Upon release, these proteins bind to Toll-like receptors (TLRs) on immune cells to trigger a signaling cascade that culminates in the activation of activator protein 1 (AP-1) transcription factors JUN, activator of transcription 2 (ATF2), and cAMP response element binding (CREB) [33]. Coincidentally, TLR signaling also activates NF-κB in immune cells that, together with the AP-1 transcription factors, triggers the production of cytokines to steer inflammation. In this respect, IL-1α is a cytokine with DAMP-like properties that is released from dying tumor cells and binds the IL-1 receptor (IL-1R) on immune cells [33,34]. Subsequently, IL-1R acts in conjunction with the TLRs to activate AP-1 and NF-κB transcription factors, culminating in IL-1α upregulation in immune cells [33]. Thus, IL-1α can potentially amplify the pro-inflammatory DAMP signaling upon its release by dying tumor cells. Other prominent cytokines that are potentially upregulated by the aforementioned transcription factors are IL-1β, IL-2, IL-6, IL-10, and TNF-α [2]. Although the current study was not designed to furnish in-depth analysis of the post-therapeutic inflammatory response induced by PDT, some interesting observations were made with respect to a small panel of cytokines.

The cytokines investigated in this study are attractants for a variety of immune cells such as neutrophils, macrophages, dendritic cells, and T-lymphocytes [35,36]. The PDT-mediated elevation of cytokine secretion may support the influx of immune cells to the treated site [37] and the orchestration of an anti-tumor immune response [17,18,19,20]. However, cytokine secretion by tumor cells is not ubiquitous and depends on the degree of photo-oxidative damage. In terms of TNF-α, which was substantially elevated up to an EMT-6 cell viability of ~20% induced with 30 nM of ZnPC in this study, the results are not ubiquitous. For example, Byun *et al.* observed no increases in TNF-α transcript and protein levels in HaCaT human keratinocyte cells treated by aminolevulinic acid PDT [38]. Another example of the differential immune response to PDT was reported by Shixiang *et al.*, who found increased levels of both TNF-α and IL-12 in rats that received an injection of PDT-treated C6 glioblastoma cell lysates treated with hematoporphyrin PDT [39]. Indeed, we also observed elevated TNF-α levels after PDT, but IL-12p70 remained unaltered. In any case, the induction of TNF-α is beneficial to PDT efficacy. TNF-α is not only a chemoattractant for immune cells that facilitate tumor cell clearance but also a strong inducer of extrinsic apoptosis and necroptosis in tumor cells [36,40]. Under conditions of oxidative stress, TNF-α can reverse the pro-survival effects of ASK-1 signaling to promote apoptosis instead [2]. Thus, an increase in TNF-α levels in combination with oxidative stress (*i.e.*, PDT) is likely to have detrimental effects on tumor cell survival [2,41]. In fact, it is not unlikely that the photosensitizer concentration-dependent reduction in post-irradiation cell viability was in part enforced by auto/paracrine TNF-α-mediated cell death.

Another important player in post-PDT immunity is CCL2, the main function of which is to promote immune cell chemoattraction to the damage site [35] and hence mediate the anti-tumor immune response after treatment. Kawczyk-Krupka *et al.* showed a reduction in CCL2 expression upon 5-aminolevulinic acid PDT of SW620 human colon cancer cells [42], an effect that was corroborated in this study with EMT-6 cells. Whereas the intensity of CCL2 immune signaling by tumor cells was proportionally reduced by photo-oxidative damage, the CCL2 signaling by macrophages was inversely proportional to tumor cell viability. Accordingly, the results suggest that the CCL2 immunomodulation is leveraged from tumor cells to macrophages with increasing PDT-induced tumor cell death.

The release of IFN-γ, IL-10, and IL-12p70 were also determined in the supernatant of PDT-treated EMT-6 cells. IFN-γ promotes antigen presentation and modulates T-cell activity [43], but its role in the anti-tumor immune response is currently elusive. Although PDT induced IFN-γ release by G422 gliomas in mice [44], our results show that the expression of IFN-γ was neither released by EMT-6 cells following PDT nor released by RAW 264.7 macrophages primed with tumor cell-derived supernatant. These results hence indicate that IFN-γ release depends on cell type and PDT regimen.

IL-12p70 is a pro-inflammatory cytokine composed of a IL-12p35 homodimer that exerts anti-tumorigenic effects by promoting cytotoxic T-cell responses [45]. IL-12 was upregulated by PDT in EMT-6 cells following porfimer sodium PDT [46] that, in light of our data, suggests that photosensitizer localization not only dictates the mode of cell death [5] but also the type of immunological signals that are elicited after PDT. IL-10 is an immunosuppressive cytokine that counteracts NF-κB and prevents the NF-κB-mediated expression of IL-6, TNF-α, and IL-12 [47]. Similarly to IL-12, IL-10 was secreted by RAW 264.7 cells after phagocytosis of Rose Bengal acetate-photokilled HeLa cells [48]. In this study, both cytokines were present at low levels in the supernatant of both irradiated EMT-6 and RAW 264.7 cells primed with EMT-6-derived supernatant. Moreover, the expression levels were unaffected by PDT or RelA knockdown, altogether indicating that IL-10 and IL-12 did not modulate IL-6 and TNF-α signaling and did not play a role in the PDT response by tumor cells or the immunogenicity of PDT-afflicted EMT-6 cells in macrophages. In line with our findings, Garg *et al.* showed that human dendritic cells do not express detectable levels of IL-10 when stimulated with hypericin PDT-treated T24 human bladder cancer cells [49]. What impact the absence of IL-12p70 release has on post-PDT adaptive immunity and anti-cancer immunity warrants further investigation.

PDT with different photosensitizers generally induces IL-6 transcription in and subsequent secretion by tumor cells [46,50,51,52], which commonly is associated with improved PDT outcome [17,53,54]. With respect to post-PDT IL-6 upregulation, Gollnick *et al.* demonstrated enhanced IL-6 mRNA expression in EMT-6 cells following PDT with porfimer sodium *in vitro* and *in vivo* [46]. In a subsequent study, the same group found that PDT with 2-(1-hexyloxyethyl)-2-devinyl pyropheophorbide-a (HPPH) caused an increase in intratumoral as well as circulating IL-6 levels in EMT-6 tumor-bearing mice [52]. Antibody-based IL-6 blocking did not significantly reduce the number of intratumoral neutrophils post-PDT. However, Cecic and Korbelik showed that anti-IL-6 antibodies significantly reduced the amount of circulating neutrophils following porfimer sodium PDT in EMT-6-bearing mice, although the effect on treatment outcome was not investigated [37]. It should be noted that exceptions have been reported for PDT-induced IL-6 signaling. For instance, Du *et al.* did not observe increased IL-6 mRNA expression in certain nasopharyngeal cancer cell lines treated by PDT [55], suggesting that PDT-induced IL-6 signaling in tumor cells is tumor type-dependent. Nevertheless, IL-6 signaling from the treated site is most likely to occur after PDT, as tumor-infiltrated macrophages, driven to the damaged tissue by various chemotactic signals [56], may initiate or take over the IL-6 signaling upon activation and positively contribute to the outcome of PDT via activation of the immune system. Corroboratively, IL-6 production and secretion was highly stimulated in macrophages by PDT-treated tumor cell supernatants in a tumor cell death-dependent manner (this study). IL-6 signaling may have been amplified by high levels of TNF-α, and possibly IL-1α, inasmuch as the stimulation of immune cells with TNF-α induces IL-6 expression through activation of NF-κB [2,33]. Similarly, the supernatant of PDT-treated human cervical carcinoma (HeLa) cells induced increased IL-6 secretion from macrophages [57]. In addition, Kushibiki *et al.* showed that the lysates of PDT-treated Lewis lung carcinomas stimulated IL-6 secretion by dendritic cells [58]. Dendritic cells are replete in solid tumors [59], where they not only mediate tumor cell death/removal by relaying immunological signals to various lymphocyte subsets (adaptive immune response) [60,61], but also contribute to immune cell chemotaxis and activation.

Despite the abovementioned findings, the mechanisms underlying IL-6-associated tumor removal [17,53,54] and adaptive immune response regulation are currently elusive and, above all, conflicting. In a study using IL-6-deficient (*IL6*^−/−^) mice, Brackett *et al.* demonstrated that IL-6 affects the levels of systemic neutrophils but not their accumulation in tumor tissue or the tumor-draining lymph node [62]. Moreover, the genetic deletion of *IL6* had no significant effect on primary anti-tumor T-cell activation post-PDT. In fact, IL-6 enhanced tumor survival post-PDT by negatively regulating anti-tumor immune memory and eliciting anti-apoptotic effects. While IL-6 may facilitate tumor cell survival and ameliorate the anti-tumor adaptive immune response, it seems to deter tumor cell proliferation, which is beneficial for PDT outcome. In the same study by Brackett *et al.*, splenocytes isolated from Colo26 tumor-bearing wild type and *IL6*^−/−^ mice that had been tumor-free for 60 days were transferred to naive immunodeficient mice. Subsequently, Colo26 tumor cells were xenografted into these mice and the formation of lung tumors was examined after 18 days. Mice that had received splenocytes from either wild type or *IL6*^−/−^ mice had significantly fewer lung tumors compared to control mice. However, mice that had received the splenocytes from *IL6*^−/−^ mice had significantly less lung tumors compared to mice that had received wild-type mouse splenocytes [62], demonstrating that the *IL6*^−/−^ splenocytes were more efficient in eliciting an anti-tumor immune response than wild type splenocytes. Thus, IL-6 exerts anti-apoptotic effects while concomitantly downmodulating anti-tumor immune memory. These biological effects of IL-6 may therefore also apply to the PDT setting. Conversely, IL-6 has been associated with proliferative signaling in both tumor cells and immune cells via binding to glycoprotein 130 and subsequent signal transducer and activator of transcription (STAT) 3 signaling [63]. On the other hand, IL-6 exerts anti-proliferative effects on endothelial cells [64]. Accordingly, PDT-induced IL-6 signaling in the tumor microenvironment might contribute to an anti-tumor innate and adaptive immune response while restraining angiogenesis. Furthermore, increased apoptosis was observed in the tumors of *IL6*^−/−^ mice compared to wild type mice following PDT, which may be linked to the expression of pro-apoptotic Bax in the tumors of *IL6*^−/−^ mice [62]. This finding is in conflict with the study of Usuda *et al.*, who showed that IL-6 overexpression sensitizes Lewis lung carcinoma cells to PDT by increased Bax expression [65]. Taken altogether, IL-6 seems to be beneficial for PDT outcome by deterring proliferation and stimulating anti-tumor immunity. However, the variance in results makes it difficult to draw definitive conclusions on the effect of IL-6 on tumors in a post-PDT microenvironment.

The discrepancies in cytokine expression profiles between the current study and some of the aforementioned investigations may be explained by differences in the types of tumor cells, immune cells, the experimental setup and PDT regimen, as well as the type of photosensitizer used. Moreover, the degree of cell death achieved in each study is also an important determinant, as cells need energy and a functional metabolic and synthetic machinery to produce and convey biochemical and biological signals. This is illustrated by the finding that extensively damaged tumor cells (60 μM ZnPC-ETL group) no longer secrete TNF-α and CCL2. Also, the intracellular localization varies per photosensitizer [5]. Oxidative damage to cytosolic components, mitochondria, or cell- and subcellular membranes will have differential effects on the mode of cell death [5], execution of survival signaling [2], and consequently the release of DAMPs to steer immune signaling [28]. This study revealed that the degree of cell destruction is proportional to macrophage activation and pro-inflammatory signaling *in vitro*. However, from the discussion it is evident that post-PDT inflammatory signaling and the consequent anti-tumor immune response as well as abscopal effects may vary in efficacy per cancer type, particularly in the *in vivo* setting.

The most important findings of this study encompass NF-κB signaling. Inhibition of NF-κB desensitized tumor cells to cell death and hypersensitized macrophages to pro-inflammatory signaling. The reduced PDT efficacy upon NF-κB knockdown may have been caused by a reduction in pro-apoptotic signaling stemming from the activated transcription factor [2]. A novel discovery was the role of tumor cell NF-κB on post-treatment immune signaling by macrophages, which led to a substantially higher release of TNF-α and IL-6 by macrophages. This response was especially prominent when PDT (60 μM ZnPC-ETLs) induced complete tumor cell death, at which point pro-inflammatory signaling by tumor cells was abrogated (TNF-α and CCL2). At this point the mechanisms that drive this response are elusive. Since there is a clear intricate relationship between NF-κB, the dying cell excretome (e.g., DAMPs, tumor-specific antigens, and cytokines), and the subsequent effect on immune cells, this mechanism should be elucidated and validated in an *in vivo* model of cancer to gauge its translational value and determine its clinical potential.

## 4. Experimental Section

### 4.1. Preparation of Zinc Phthalocyanine Liposomes

Cationic liposomes composed of dipalmitoylphosphocholine, dicarbimoyl cholesterol, cholesterol, and distearoylphosphoethanolamine-polyethylene glycol (66:25:5:4 molar ratio) were prepared by the lipid film hydration technique as described [6,8]. ZnPC (Sigma-Aldrich, St. Louis, MO, USA) was added to the organic phase at a 0.003 molar ratio to lipids. The liposomes were taken up by cultured tumor and human umbilical vein endothelial cells *in vitro* [24] and were developed for targeting to the tumor vasculature *in vivo* in accordance with [66]. These liposomes were therefore termed zinc phthalocyanine-encapsulating endothelium-targeted liposomes (ZnPC-ETLs). All lipids were from Avanti Polar Lipids (Alabaster, AL, USA), with the exception of distearoyl-phosphoethanolamine-polyethylene glycol (Sigma-Aldrich).

### 4.2. Cell Culture

The EMT-6 cells were cultured in RPMI 1640 (Gibco/Life Technologies, Carlsbad, CA, USA) culture medium containing phenol red and l-glutamine, supplemented with 10% (*v*/*v*) fetal calf serum (FCS) (Lonza, Walkersville, MD, USA), penicillin (100 U/mL, Lonza), streptomycin (100 μg/mL, Lonza), and (1 × 10^−^^6^)% (*v*/*v*) β-mercaptoethanol and cultured in T75 flasks under standard culture conditions (37 °C, 21% oxygen, 74% nitrogen, 5% carbon dioxide). Cells were passaged once a week at a 1:20 ratio following Accutase digestion (Merck Millipore, Darmstadt, Germany) and received fresh medium at 3–4 day intervals.

The RAW 264.7 murine macrophage cell line was cultured in Dulbecco’s modified Eagle’s medium (DMEM) (Gibco/Life Technologies) containing phenol red and l-glutamine and supplemented with 10% FCS and 1% penicillin/streptomycin in T75 flasks under standard culture conditions. Cells were typically passaged once a week at a 1:20 ratio following harvesting with a cell scraper (NUNC, Thermo Scientific, Tewksbury, MA, USA) and cells received fresh medium every 2–3 days.

### 4.3. Photodynamic Therapy

PDT was performed on EMT-6 cells that had been seeded in 24-well plates (1.5 × 10^5^ cells per well) and cultured to subconfluence for 24 h. When indicated, cells were pretreated with 1 μg/mL LPS (Sigma-Aldrich) during the first 20 h following seeding, after which cells received fresh medium 4 h prior to further treatment. Immediately prior to PDT, the cells were photosensitized for 1 h with 0–60 μM ZnPC-ETLs (final lipid concentration) in DMEM devoid of FCS and phenol red. Cells were washed once with 1 mL PBS and received 0.3 mL DMEM devoid of FCS and phenol red. Subsequently, cells were irradiated at a cumulative radiant exposure of 15 J/cm^2^ (500 mW, 60 s, 2 cm^2^ spot size) using a 671-nm solid-state diode laser (CNI, Changchun, China). Viability was assayed using the water-soluble tetrazolium-1 (WST-1) method (Roche Diagnostics, Basel, Switzerland) [6] after 24 h of incubation under hypoxic culture conditions (37 °C, 95% nitrogen_,_ 5% carbon dioxide, Linde Gas, Schiedam, The Netherlands) to simulate vascular shutdown that ensues PDT *in vivo* [67]. The mode of cell death was determined using the Vybrant Annexin V-AlexaFluor488/propidium iodide apoptosis detection kit (Molecular Probes, Eugene, OR, USA) [6].

The medium of PDT-subjected EMT-6 cells was collected and centrifuged for 10 min at 800× *g* (4 °C). The supernatant was aspirated and the pellet containing cell debris was dissolved in 0.1 mL Annexin-V binding buffer. Cells were subsequently stained for flow cytometric analysis (FACSCanto II, Becton Dickinson, Franklin Lakes, NJ, USA) as described in [6].

### 4.4. Stimulation of RAW 264.7 Macrophages

RAW 264.7 macrophages were seeded in 24-wells plates at a density of 1.5 × 10^4^ cells/well and typically grew to subconfluence in 24 h. Cells were stimulated with either 1 μg/mL lipopolysaccharide (LPS, positive control) in 0.3 mL serum and phenol red free DMEM (negative control) or medium harvested from the EMT-6 cells (0.3 mL). RAW 264.7 cells were incubated for 24 h, after which the media was collected and cells were analyzed for viability using the WST-1 assay. Culture media were immediately analyzed for nitrite content or stored at −20 °C for cytokine analysis. Macrophage activation was determined by measuring the nitrite concentrations in the medium using the Griess reagent system (Promega, Madison, WI, USA). Concentrations were corrected for the mean viability of the macrophages per treatment group.

### 4.5. Cytokine Detection

Cytokine secretion by EMT-6 and RAW 264.7 macrophages was analyzed with a cytometric bead array mouse inflammation kit (BD Biosciences, Franklin Lakes, NJ, USA) according to the manufacturer’s protocol. The cytokines assayed included TNF-α, CCL2, IL-6, IL-10, IL-12p70, and IFN-γ. The limits of detection for TNF-α, CCL2, IL-6, IL-10, IL-12p70, and IFN-γ were 7.3, 52.7, 5.0, 17.5, 10.7, and 2.5 pg/mL, respectively. 

### 4.6. Transfections

EMT-6 cells were transfected with RelA siRNA (RSS317831 stealth RNA, Life Technologies) or with low GC-content control siRNA (Life Technologies). EMT-6 cells were seeded in 24-wells plates (5 × 10^4^ cells/well) and allowed to adhere to the plate for 2 h. Subsequently, cells were washed in 0.5 mL of PBS and received 0.4 mL of fresh supplemented OptiMEM medium (Gibco). Separately, 1 μL of lipofectamine and 2.5 μL of siRNA (20 nM) were mixed in separate tubes with 50 μL of supplemented OptiMEM per sample. After 15 min incubation, the tubes were combined and incubated for an additional 15 min. Afterwards, 100 μL of lipofectamine-siRNA solution was added to the sample wells. Cells were incubated for 24 h in supplemented OptiMEM prior to further experiments. The siRNA had a knockdown efficiency of ~75% as determined by qRT-PCR [68].

### 4.7. Statistical Analysis

Statistical analyses were performed in GraphPad Prism 6.0 software (GraphPad Software, San Diego, CA, USA). Unless when indicated otherwise, all data was analyzed using a Kruskal–Wallis test and a Dunn’s *post-hoc* test for multiple comparisons. Larger data sets (*N* = 6) were assessed for normality using a Kolmogorov–Smirnov test, and analyzed using a one-way ANOVA and a Sidak’s *post-hoc* test when the data was normally distributed. Levels of significance are reflected by single (*p* < 0.05), double (*p* < 0.01), triple (*p* < 0.005), or quadruple signs (*p* < 0.001). Significant differences *versus* the corresponding control group are indicated with a pound sign, other relevant intergroup differences are indicated with asterisks. IC_50_ values were determined using the least squares fit. IC_50_ values were compared using a sum-of-squares *F*-test.

## 5. Conclusions

NF-κB has a bivalent role following PDT that appears to be ambivalent. On the one hand, NF-κB promotes cell demise, which is required for acute tumor eradication following PDT. On the other hand, NF-κB reduced the immunogenicity of PDT-subjected dead and dying tumor cells, which is a prerequisite for long-term tumor eradication. Thus, when NF-κB is considered as a pharmacological intervention target to improve PDT outcome, it is advisable to take its effects on the immune system into consideration.
